# Integrating transcriptomic analysis to explore biomarkers and therapeutic targets for rheumatoid arthritis: a case-control and systems biology approach

**DOI:** 10.3389/fbinf.2026.1891543

**Published:** 2026-10-05

**Authors:** S. Aswini, S. Mohamed Muzammil, N. Raja, S. Asha Devi

**Affiliations:** 1 School of Bio Sciences and Technology, Vellore Institute of Technology, Vellore, Tamil Nadu, India; 2 Padmavathy Rheumatic Care Centre, Vellore, Tamil Nadu, India

**Keywords:** differentially expressed genes, IFNG, IL2RG, inflammatory cytokines, rheumatoid arthritis, synovial macrophages

## Abstract

**Background:**

Rheumatoid Arthritis (RA) is a highly variable and unpredictable autoimmune disease, relying solely on physical symptoms often leads to delayed treatment and irreversible joint destruction. Therefore, identifying biomarkers in RA is necessary to bridge the gap between a patient’s unique biological profile and successful clinical management. This study aims to identify distinct molecular signatures in RA patients by integrating systems biology and case-control methodologies.

**Methods:**

Transcriptomic datasets from various sources specifically synovial tissues, macrophages, blood, T-cells, natural killer T-cells, natural killer cells, neutrophils, and monocytes were analysed. Differentially Expressed Genes and functional enrichment pathways were identified, followed by Weighted Gene Co-expression Network Analysis to pinpoint hub genes. Machine learning models were used to extract consensus biomarkers. Two of the predicted biomarkers IL2RG) and Interferon-Gamma (IFNG) were further validated using an Enzyme-Linked Immunosorbent Assay (ELISA) in RA patient serum samples.

**Results:**

The analysis revealed that synovial tissues and synovial macrophages exhibited pronounced transcriptomic expression changes. Six consensus biomarkers such as IFNG, PTEN, IL2RG, CDKN2D, VEGFA, and CDKN1A were computationally prioritized in synovial macrophages. Among these, IL2RG and IFNG were selected for preliminary experimental validation by ELISA. IL2RG concentrations were significantly higher in RA patients (1.34 ± 0.11 ng/mL) than in healthy controls (0.83 ± 0.07 ng/mL), while IFNG levels showed only a marginal increase (1.91 ± 0.06 pg/mL) compared to controls (1.69 ± 0.06 pg/mL).

**Conclusion:**

By integrating multi-tissue transcriptomic profiling with machine learning, this study mapped critical molecular changes in RA and prioritized six consensus biomarkers within synovial macrophages. Experimental validation confirmed that IL2RG and IFNG are significantly elevated in RA patients compared to healthy controls. These findings offer a promising, minimally invasive molecular markers for diagnosis and personalized clinical management in RA.

## 1 Introduction

Rheumatoid Arthritis (RA), an autoimmune condition defined as chronic joint inflammation and progressive damage, ultimately leading to pain and long-term disability (Taylor, 2020). The global prevalence of RA is nearly 1%, while women being diagnosed at twice the rate than men. RA risk is driven from a combination of genetics and environment factor, that together triggers immune dysfunction. Immune cells, predominantly T-cells, infiltrate the synovium, triggers the release of pro-inflammatory cytokines such as Tumour Necrosis Factor (TNF) -α, IL (Interleukin)*-*1, and IL-6, which recruit macrophages and B-cells in RA. Macrophages promote synovial hyperplasia and pannus formation via increased inflammation, while B-cells produce autoantibodies that increases joint damage. RA synovitis exhibits significant heterogeneity, with distinct cellular and molecular signatures forming different synovial pathotypes, including lymphoid, myeloid, pauci-immune, and fibroid variants. These variants differ in their immune cell composition and architectural organization, creating clinical challenges for both diagnosis and therapeutic intervention (Ouboussad et al., 2019; Zhao et al., 2021).

Advanced therapeutics such as TNF inhibitors improve the signs and symptoms of disease, inhibit progression of joint damage, and restore physical mobility in patients (Hughes, 2009). Targeted therapies such as methotrexate and biologics serve as first-line treatment for RA management (Emery et al., 2008; Sparks, 2019). These, along with emerging cell therapies, have facilitated a range of multi-drug regimens (Shams et al., 2021). Despite recent progress in biological and pharmacological therapies, a significant proportion of RA patients experience inadequate results or unendurable side effects, resulting in unchecked disease progression (Bykerk et al., 2012). Hence, understanding pathophysiology of RA and developing more accurate biomarkers are critical for early diagnosis and therapy.

In recent study using bioinformatics and datasets like GSE55235, Differentially Expressed Genes (DEGs) linked to regulated cell death pathways specifically ferroptosis and cuproptosis in RA patients compared to healthy controls. Through functional enrichment, network analysis, and validation, the studies highlight specific hub genes and signaling pathways that can serve as crucial diagnostic biomarkers and novel therapeutic targets for RA (Li et al., 2023; 2025). Similarly, DNA Microarray technology is preferred to identify the genes associated with specific disease. Various microarray studies have identified putative gene signatures responsible for aetiology of RA. For the current study, DEGs from different array samples, namely, synovial tissue, peripheral blood, and various immune cells, including, T cells (CD4^+^, CD8^+^), macrophages, Natural Killer T cells (NKT cells), Natural Killer (NK) cells, neutrophils, monocytes, and CD14^+^ cells were chosen. Out of these sample types, synovium samples had significant expression patterns compared to other cell types and sources, therefore synovium samples were chosen for further investigation. Thus, focus on synovial tissue and synovial macrophages gives precise insights, including functional enrichment and pathway analysis, co-expression analysis, and biomarker discovery using Weighted Gene Co-expression Network Analysis (WGCNA). Further, the predicted biomarkers such as Interferon -Gamma (IFNG) and Interleukin-2 Receptor Gamma (IL2RG) were validated using Enzyme-Linked Immune Sorbent Assay (ELISA). Thus, this study investigates RA-associated molecular signatures in synovial tissue, particularly synovial macrophages using transcriptomics analysis.

## 2 Materials and methods

### 2.1 Data retrieval

The gene expression datasets intended for RA are retrieved from NCBI’s Gene Expression Omnibus (GEO) database (Barrett et al., 2013). “Rheumatoid Arthritis” was used as a search term, restricting the organisms to “*Homo sapiens*”, entry type to “Series” and study type to “Expression profiling by array”. A total of 198 results were obtained as of October 2024. Further, based on the study summary and overall experiment design, the datasets were scrutinized. The datasets which included the following information were selected for further analysis. The Inclusion criteria were that datasets must contain both diseased and healthy control samples. Datasets containing healthy controls were only considered. Samples undergone any drug treatment; samples with unclear annotation, duplicated or missing expression were not included. The entire screening process identified around 18 GEO datasets. For further study, the gene expression profiles and related clinical annotation data were retrieved. Supplementary Table S1 provides comprehensive details on the datasets utilized in the research.

### 2.2 Data processing

The downloaded gene expression profiles are processed using various packages in R. The probes not associated with any known genes were removed from further analysis. If multiple probes match a single gene, the probes with the highest Interquartile Range (IQR) were selected. Quantile normalization was performed using Normalize Between Arrays. To eliminate batch effects multiple datasets were merged together by ComBaT method using SVA package in R (Leek et al., 2012). The DEG with a threshold of logFC >1 and adjusted *p* < 0.05 were identified using Limma package in R (Ritchie et al., 2015). Several datasets were combined using the Robust Rank Aggregation (RRA) technique to produce a ranked gene list using the RRA package in R (Kolde et al., 2012).

Principal Component Analysis (PCA) was carried out to assess the impact of batch effects before and after the batch correction using prcomp () function in R. The data samples were visualized based on first two principle components. PCA plots were generated to analyse samples clustering based on both batch and biological condition. Principal variance component analysis (PVCA) was carried out to further quantify the relative contribution of batch effects and biological conditions. The analysis was performed using pvca package considering both the batch and disease conditions as explanatory variables. PVCA analysis was performed before and after batch correction to evaluate the effectiveness of batch correction while maintaining the biological relevance.

### 2.3 Functional enrichment analysis

The Gene Ontology (GO) which includes Biological Process (BP), Molecular Function (MF), and Cellular Components (CC) along with the enriched KEGG (Kyoto Encyclopaedia of Genes and Genomes) pathways of the DEG were analysed using clusterProfiler (Yu et al., 2012), org. HS.e.g.,.db, ggplot2, and enrichplot packages in R. The significantly enriched GO and pathways were screened at corrected *p-value* < 0.05.

### 2.4 Weighted gene co-expression network analysis

The WGCNA method is not only used to construct gene networks but also to detect gene modules as well as to identify the hub genes within modules that show crucial relationships with the disease trait (Langfelder and Horvath, 2008). The batch-corrected expression matrix was used as input for WGCNA. The power of β = 8 and 7, a scale-free *R* ^2^ = 0.981 and 0.929 for synovial tissue and synovial macrophage datasets were used respectively as soft threshold parameters to confirm a signed scale free co-expression gene network. The minimal module size was set to 20. The modules with a high correlation with disease traits were chosen for further analysis. We further identified crucial genes based on Gene Significance (GS) and Module Membership (MM).

The genes with GS and MM thresholds were obtained for the identified modules. The protein partners of the module genes were retrieved from STITCH database and the confidence score was set to 0.7 (Kuhn et al., 2008). The mapped proteins were visualized in Cytoscape 3.10.2 and the network statistics of each module were computed using Network Analyst plugin of Cytoscape (Paul Shannon et al., 1971).

### 2.5 Biomarker identification and validation

Two datasets of synovial macrophages, GSE10500 and GSE97779, were utilized for biomarker discovery and classifier development due to limited availability of synovial macrophage transcriptomic data. Module genes identified through the WGCNA method is used as initial space for machine-learning analysis. The expression data were filtered to include only these module genes and hypothesized to play a crucial role in RA pathogenesis based on their co-expression patterns and established biological relevance.

The macrophage dataset GSE97779 was used as training dataset. Model performance was assessed using one non-independent synovial macrophage evaluation dataset and two additional external evaluation datasets. GSE10500 (test set1) was considered a non-independent evaluation set because it contributed to biomarker discovery and was therefore not treated as an independent validation cohort. GSE49604 (test set2) was used as an external synovial macrophage evaluation set, and the combined synovial tissue expression dataset (GSE12021, GSE1919, GSE55235, GSE554587 and GSE77289 – test set3) was used as a cross-tissue exploratory evaluation set (Table 1). Given the small sample size, 5-fold cross validation (k = 5) was employed for model tuning and performance assessment. Two classifiers were trained on the module gene expression data: a Support Vector Machine (SVM) with a radial kernel with a cost value (c) of 10 and a Random Forest (RF) with a Number of trees equal to 500. Optimization of hyperparameter was tuned using grid search within a cross-validation framework. Given the small sample size, the resulting performance estimates were interpreted cautiously and considered exploratory rather than definitive.

**TABLE 1:** Sample size and class distribution of the datasets used for classifier building.

Dataset	Dataset role	Sample type	Number of RA samples	Number of normal samples
GSE97779	Training	Synovial macrophages	9	5
GSE10500	Test set 1 (non-independent evaluation set)	Synovial macrophages	5	3
GSE49604	Test set 2 (external evaluation set 1)	Synovial macrophages	2	6
GSE12021 GSE1919 GSE55235 GSE55457 GSE77298	Test set 3 (external and cross-tissue evaluation set 2)	Combined synovial tissue	78	19

To prioritize candidate biomarkers SVM-based Recursive Feature Elimination (RFE), and RF feature-importance scores were used. From the WGCNA red module, 88 genes with both GS > 0.9 and MM > 0.9 were selected as the initial candidate feature set for machine-learning analysis. SVM-RFE was then applied to rank and reduce the candidate genes according to their contribution to classification performance. In parallel, RF feature importance was calculated using the mean decrease in Gini index. Candidate biomarkers were defined as consensus genes only if they were retained by SVM-RFE and ranked among the most important RF features. The final six consensus biomarkers represented the intersection between the SVM-RFE-selected genes and RF-prioritized genes.

Model performance was evaluated using multiple classification metrics which includes accuracy, sensitivity, specificity, precision, F1-score and area under the receiver operating characteristic curve (AUC). Confusion matrix was generated for the training and test sets to analyse the class-specific performance. Receiver operating characteristic (ROC) curves were constructed, and AUC values were estimated using the pROC package in R. Where applicable, 95% confidence intervals were calculated for AUC and classification performance metrics to reflect uncertainty associated with the limited sample size. The e1071, randomForest, caret, and pROC R packages were used for biomarker identification, classifier construction, and ROC curve generation and model validation.

Because of the limited availability of synovial macrophage datasets, the datasets used for feature selection and model development were not completely independent. Hence, the model-building strategy is likely to introduce information leakage and increase overfitting. Hence, the machine-learning analysis should be interpreted as exploratory approach for prioritizing candidate biomarkers rather than definitive diagnostic model.

### 2.6 Quantification of IL2RG and IFNG levels in serum samples

3 mL of blood samples were collected from 113 RA patients and 104 healthy controls at Sri Narayani Hospital and Research Centre, Vellore, India. RA patients were selected based on the American College of Rheumatology and the European League Against Rheumatism. Primary inclusion criteria confirmed an RA diagnosis and excluded co-existing autoimmune or systemic inflammatory conditions. In terms of demographic breakdown, the RA patients include 25 males and 88 females, while the healthy control group comprises 20 males and 84 females. The mean age was 50 ± 11 years for the RA patients and 40 ± 15 years for the healthy controls. To minimize the potential confounding effect of age differences between the two groups, a significant portion of the healthy control samples was collected from individuals aged between 45 and 50 years. Serological evaluation among the RA cohort reveals that ESR positivity 67.26%, IgG-RF positivity 81.42%, anti-CCP positivity 71.68%, and CRP positivity 84.07%. These specific inflammatory and autoantibody markers are not applicable for the healthy controls. Regarding therapeutic interventions, none of the RA patients are actively undergoing treatment, for NSAID and DMARD. Clinical disease activity assessments showed a mean DAS28 score of 3 ± 1 for the RA group, contrasting with a baseline score of 0 ± 0 for the healthy controls. This study was approved by Institutional Ethical Committee (IEC/IRB No. 29/08/07/2022) and all participants provided their informed consent before the sample collection. Out of six predicted RA biomarkers through computational approach, we selected IFNG and IL2RG for experimental validation due to their high GS, MM, and strong relevance to RA pathology. The non-haemolyzed serum was separated from the blood samples after centrifuging at 4,500 rpm for 10 min at 4 °C. The serum was instantly transferred to a sterile tube and stored at −20 °C to prevent degradation. Further, the concentrations of IL2RG and IFNG in serum samples were quantified following the manufacturer’s protocol of human IL2RG and IFNG ELISA kits (CUSABIO® catalog number: CSB-EL011651HU and CSB-E04577h-IS) and measured the absorbance at 450 nm using a Bio-Rad xMark™ ELISA plate reader. Samples were duplicated to calculate the unknown IL2RG and IFNG concentrations against the standard curve.

### 2.7 Statistical analysis

Statistical analysis was performed using GraphPad Prism 9.0, and significance between healthy controls and RA patients was determined using unpaired Student’s t-test. The differences in biomarker (IL2RG and IFNG) expression levels were represented as mean ± SD. A *p-*value <0.05 was considered statistically significant for unadjusted comparisons. To account for multiple comparisons across the two validated markers, a Bonferroni-adjusted significance threshold of α = 0.025 was applied.

## 3 Results and discussion

### 3.1 Transcriptomic changes in immune cells associated with RA

From 18 datasets analysed, DEGs across various tissues and immune cell types were identified in RA samples when compared to healthy controls (Supplementary Material 1). We obtained the DEGs from each study for comparison between the datasets (Figure 1). When comparing the number of DEGs across different groups, synovial tissues exhibited the highest number of DEGs, followed by macrophages and CD4^+^ T cells (Supplementary Table S2). Further, when we explored the overlap of DEGs between these groups, it was observed that DEGs were regulated in both directions, not only between different datasets but also across cell types. Additionally, some DEGs exhibited opposite regulation within samples from the same cell type. It is important to note that not all overlapping DEGs were consistently present across all datasets for a given cell type. The largest overlap was observed between synovial tissue and macrophages (n = 439), followed by macrophages and CD4^+^ T cells (n = 195), and synovial tissue and CD4^+^ T cells (n = 193). Notably, 45 DEGs were shared among synovial tissue, macrophages, and CD4^+^ T cells. Interestingly, *Matrix Metalloproteinases* (*MMPs*) were the only gene upregulated in the blood, synovial fluid, and CD4+T cells, but it was downregulated in macrophages. MMPs are Zn^2+^-dependent endopeptidases and play a key role in the degradation of Extra-Cellular Matrix (ECM) components (Stojanovic et al., 2023). MMP-9 contributes to the survival, proliferation, migration, and invasion of RA synovial fibroblasts, with pronounced effects. In addition to breakdown of denatured collagen, MMP-9 also activates other MMPs, particularly proMMP-13 collagenase, the main enzyme involved in cartilage erosion in both RA and Osteoarthritis (OA) (Bian et al., 2023; Takaishi et al., 2008; Xue et al., 2014). Synovial macrophages are situated within a highly specialized microenvironment characterized by chronic inflammation, and hypoxia, and they are in constant interactions with immune cells, fibroblasts, and components of the ECM. These conditions may contribute to the distinct regulation of *MMP9* expression when compared to circulating macrophages from the blood. Over the period, synovial macrophages undergo differentiation and respond to the local inflammatory environment, leading to alterations in their gene expression profile, including the downregulation of *MMP9* (Udalova et al., 2026). *Secretory Leukocyte Protease Inhibitor* (*SLPI*) and *IL-2RB* genes were shared by the synovial fluid, blood, and macrophages. SLPI is upregulated in blood and macrophages and downregulated in synovium. SLPI is an endogenous potent protease inhibitor that is critical for the maintenance of balance against the proteases in the joint. SLPI prevents tissue destruction by maintaining homeostasis at barrier tissues. The arthritic joints lose their ability to sequester SLP1 from the blood, as SLP1 is not produced in the tissue joints (Mongkolpathumrat et al., 2024). *(IL2RB)* is downregulated in the blood samples while upregulated in synovium and macrophages. The IL2RB*,* a constituent of the high affinity IL-2 receptor transmits IL-2 signals. This signal is important to maintain a balance between immune tolerance and inflammation. The downregulation of *IL2RB* gene in blood indicated systemic immune suppression or Treg deficiency in circulation, while upregulation of *IL2RB* in synovium reveals an overactive immune system as well as a proliferation of effector T-cells and macrophages within the joints (Van Amelsfort et al., 2004). Previous studies have reported that mutation in *IL-2RB* gene leads to immunodeficiency associated with autoimmunity (Akhmed and Lichtenstein, 2009; Ruyssen-Witrand et al., 2014). The *Golgin A2* (*GOLGA2*) gene is upregulated in synovium and monocytes, but it is downregulated in CD4 - T cells. The upregulation of GOLGA2 in synovium facilitates secretion of pro-inflammatory mediators and matrix-degrading enzymes in Golgi complex, which may lead to synovial hyperplasia and inflammation in RA (Gambari et al., 2020; Mărunţelu et al., 2024). Similarly, downregulation of *GOLGA2* gene in CD4^+^ T cells leads to altered immune responses in RA. *Proto-oncogene, Serine/Threonine Kinase-1*(*PIM1*) gene is upregulated in synovium and downregulated in macrophages and monocytes. *PIM1* gene regulates cell proliferation, migration and synthesis of matrix metalloproteins, which are the key factors required for joint destruction. Thus, enhanced expression of *PIM1* gene in RA patients may lead to synovial hyperplasia and aggression (Ha et al., 2019) which is proved by *PIM1* gene knockdown study (Baek et al., 2024). Similarly, Cystatin 7 (CST7) is a cathepsin C-directed cysteine protease which is upregulated in synovium, macrophages, and monocytes. CST7 degrades the proteins in the extracellular matrix. Increased expression of *CST7* in this cell facilitates pathogenesis of RA by degrading the cartilage components, thus leading to joint damage and inflammation condition (Sawyer et al., 2021).

**FIGURE 1:**
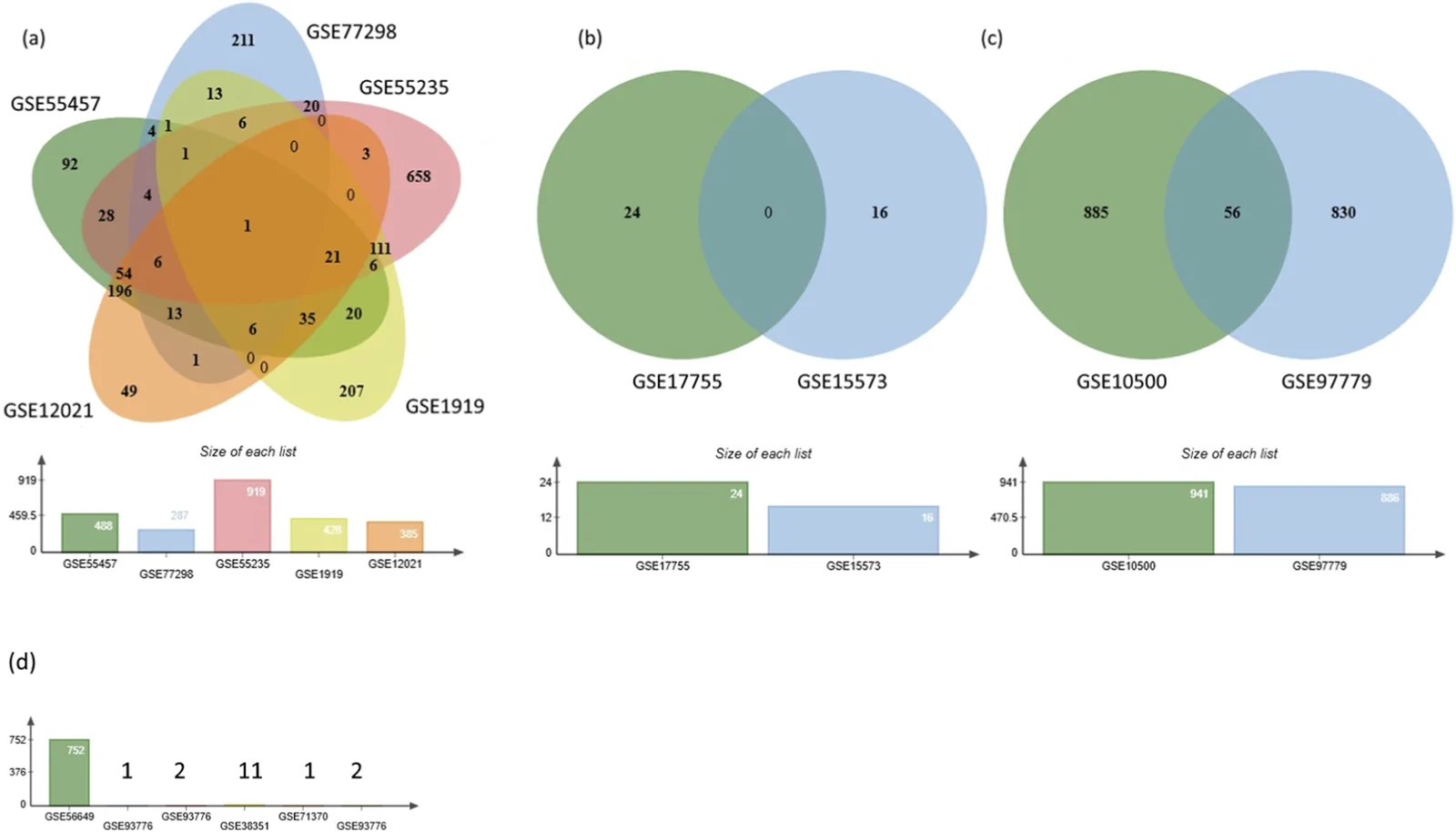
DEGs and comparative analyses of common DEGs in RA tissues. Venn diagram representing the comparison of DEGs among **(a)** Synovial tissues with RA, **(b)** Synovial macrophages, and **(c)** Peripheral blood. **(d)** The distribution of DEGs in CD4^+^ T cells, NKT cells, NK cells, Monocyte Cells, CD14^+^ cells, and CD8^+^ T cells.

### 3.2 Functional enrichment analysis of the DEG–gene ontology analysis

DEGs in synovium and synovial macrophages were investigated for their role in biological functions and pathways. These were particularly chosen as we observed a higher overlap of DEGs within similar dataset categories. The top ten enriched GO of synovium were selected based on adjusted *p*-*value < 0.05* (Figure 2). Upregulated DEGs in synovium revealed enrichment in immune-mediated processes within BP, such as immune regulation, activation, and signaling. Similarly, the MF related to immune activities, such as antigen binding, immune receptor activity, and cytokine/chemokine receptor activity were noticed. The upregulated BP and MF are associated with immune response regulation, signaling, and antigen processing revealing that synovium is not only a passive site of inflammation but also actively participates in immune signaling and coordination (Jang et al., 2022). In CC analysis, DEGs were enriched in immune related structures like, plasma membrane, endocytic vesicles, and immunoglobulin complexes.

**FIGURE 2:**
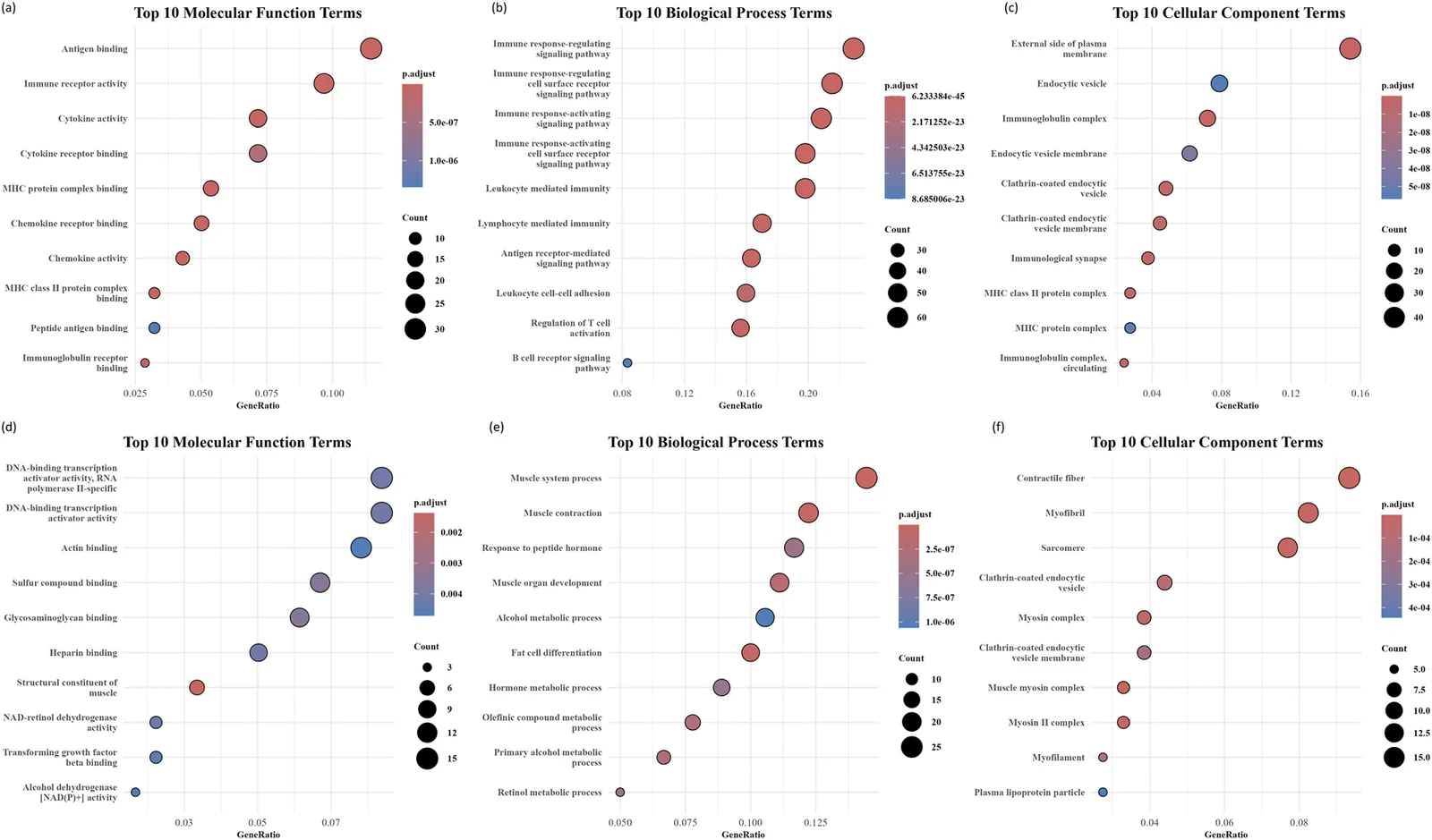
Functional Enrichment analysis of DEGs from Synovial tissues. **(a–c)** Enriched GO terms of upregulated DEG and **(d–f)** Enriched GO terms of downregulated DEG.

The CC reveals that DEG significantly contribute to chronic joint inflammation and immune-mediated disorders. Downregulated DEG revealed muscle-related BPs, including muscle contraction, development and function, furthermore CC terms were predominantly linked to muscle structures specifically contractile fibers, myofibrils, sarcomeres, and muscle myosin complex (Huffman et al., 2017). Conversely, GO analysis highlighted a significant decrease in muscle-related processes among the downregulated DEGs. The enrichment in muscle related pathways highlights that cells are shifting away from their supportive roles in muscle function. Downregulation of genes reflects pathophysiological shift in which immune activation is prioritized over muscle function. This shift can lead to the muscle weakness commonly found in inflammatory joint diseases. GO analysis of DEGs in synovial macrophages indicates a upregulated immune response, specifically involving the activation and regulation of signalling pathways, defence response against pathogens, and increased leukocyte migration and chemotaxis (Figure 3). Enriched MF terms include enzyme inhibitor activity, regulation and inhibition of peptidase and endopeptidase activities, as well as heat shock protein and calcium-dependent protein binding. Under CC category, the upregulated DEGs are linked with focal adhesion, cell-substrate junctions, and the external side of the plasma membrane. The analysis highlights the pivotal contribution of synovial macrophages to immune modulation within the joint, specifically, through pathways involved in immune cell migration, pathogen defence, and intercellular signaling. The enrichment in enzyme inhibitor and regulatory functions suggests that synovial macrophages appear to control the proteolytic activity, helping to balance joint tissue remodelling and the body’s inflammatory response. Macrophages use focal adhesions and cell-substrate junctions to stay stable and properly positioned within inflamed synovial tissue. The breakdown of the synovial macrophage barrier during inflammation allows invasion of activated fibroblasts and infiltrating of immune cells. This process triggers proliferation of pathogenic fibroblasts and Mer tyrosine kinase macrophages within sub-lining layer, establishing a pro-inflammatory environment that sustains infiltration and survival of inflammatory cells. The failure of pathogenic fibroblasts to respond to anti-inflammatory signals from macrophages may lead to chronic inflammation (Kemble and Croft, 2021). Meanwhile, the downregulated DEGs involve in toxin and neuroinflammatory responses, and MF shows enrichment of endopeptidase, peroxidase, and oxidoreductase functions. The CC terms were related to cytoplasmic and secretory vesicles, along with lysosomal membranes. Downregulation of genes involved in inflammation indicates a diminished immune response within the synovial environment. Subsequently, decreased expression of DEGs linked to vesicular and lysosomal components suggests a decline in cellular degradation and secretion. These shifts are essential for restoring tissue homeostasis by supressing excessive inflammatory activity. Under chronic inflammatory condition continuous downregulation of these processes will weaken the tissue’s ability to maintain oxidative damage and remove harmful substrates, thus leading to sustained inflammation and tissue dysfunction (Hitchon and El-Gabalawy, 2004).

**FIGURE 3:**
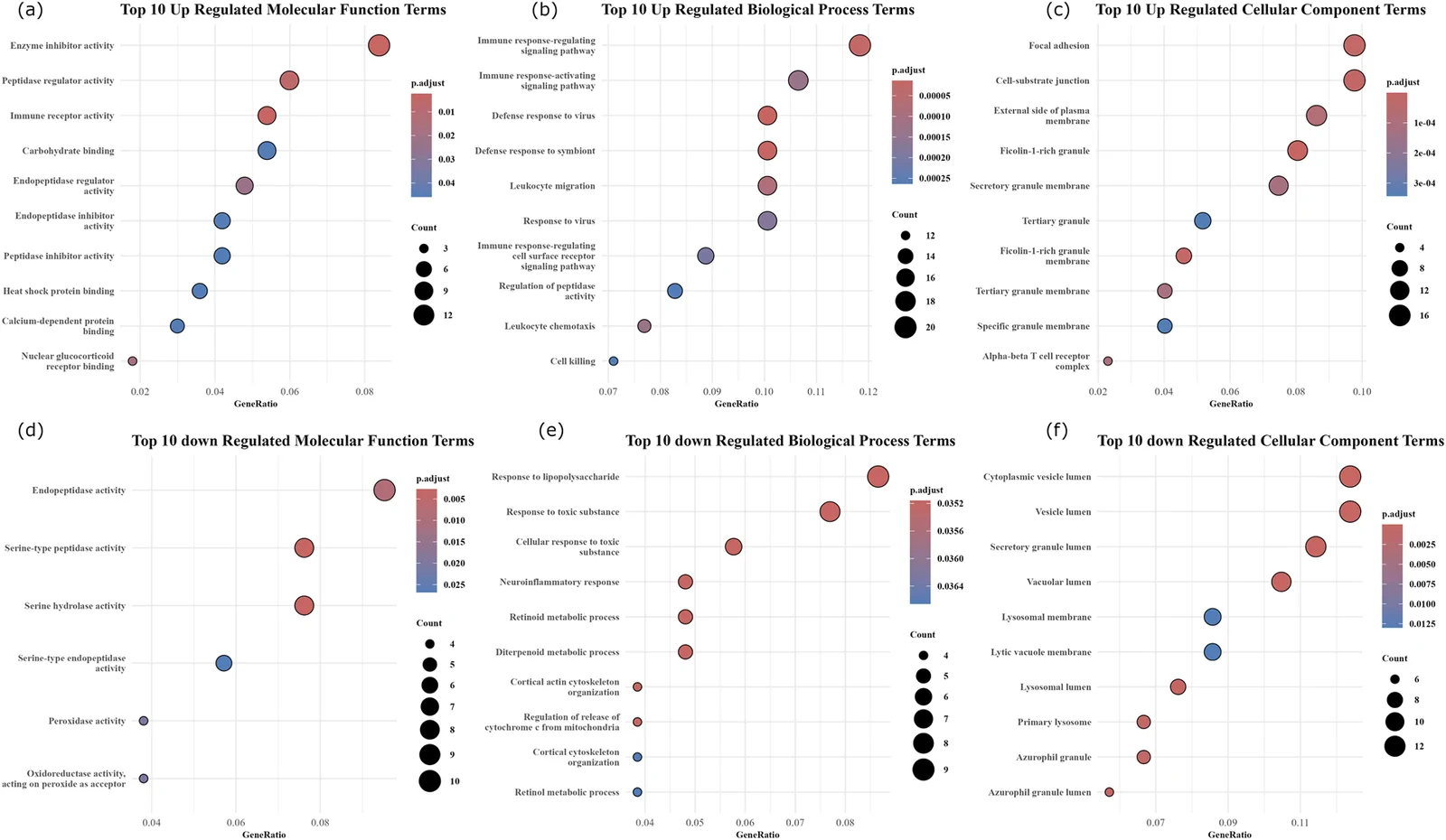
Functional Enrichment analysis of DEGs from Synovial macrophages. **(a–c)** Enriched GO terms of upregulated DEG and **(d–f)** Enriched GO terms of downregulated DEG.

### 3.3 Functional enrichment analysis of the DEG–pathway analysis

Pathway analysis of DEGs in both synovium and macrophages (Figures 4, 5) showed various upregulated and downregulated pathways. The synovial tissue transcriptome shows that the upregulated DEGs were enriched in immune-related pathways, including cytokine-cytokine receptor interactions, chemokine signaling, and RA-specific pathways. Upregulated DEGs in macrophages are linked to key pathways like Phosphoinositide-3 Kinase (PI3K) - Protein Kinase B (AKT) signaling, focal adhesion, and Mitogen-Activated Protein kinase (MAPK) signaling. Similarly, transcriptomic analysis of downregulated genes revealed that synovial tissue associated with pathways related to muscle cell cytoskeleton structure, dilated cardiomyopathy, and Peroxisome Proliferator Activated Receptor (PPAR) signaling, whereas macrophages exclusively showed downregulation within the lysosome pathway. Synovial tissue actively fuels chronic joint inflammation in RA by driving key cytokine and chemokine signaling networks. Within the inflamed synovial tissue macrophages uses PI3K/AKT and MAPK signaling to stay alive, move around, and control the immune response. The PI3K/AKT pathway plays a central role in both the onset and severity of RA. This pathway influences the proliferation of fibroblast-like synoviocytes and the secretion of critical pro-inflammatory mediators (IL-1β, IL-6, IL-17, IL-21, IL-22, and TNF-α) which are the crucial contributors for the pathological changes in RA (Dinesh and Rasool, 2018; Ding et al., 2023; Kwok et al., 2012; Mitra et al., 2012; Tsai et al., 2017). Increased activity of MAPK signaling may trigger cartilage destruction and inflammatory hyperplasia (Ding et al., 2023; Sujitha and Rasool, 2017). Additionally, the downregulated pathways showed unique tissue-specific functions. In the synovium, the downregulation of muscle-related pathways suggests a decline in structural maintenance towards immune system prioritization. This may lead to muscular and structural degradation observed in chronic inflammation. Downregulation of lysosome pathway within synovial macrophages suggests impaired degradative capacity, which may interfere with antigen processing and the effective resolution of inflammation. Overall, these pathway regulations reveal the complex interactions between immune activation and tissue remodeling, clarifying the drivers of chronic inflammation in joint disease.

**FIGURE 4:**
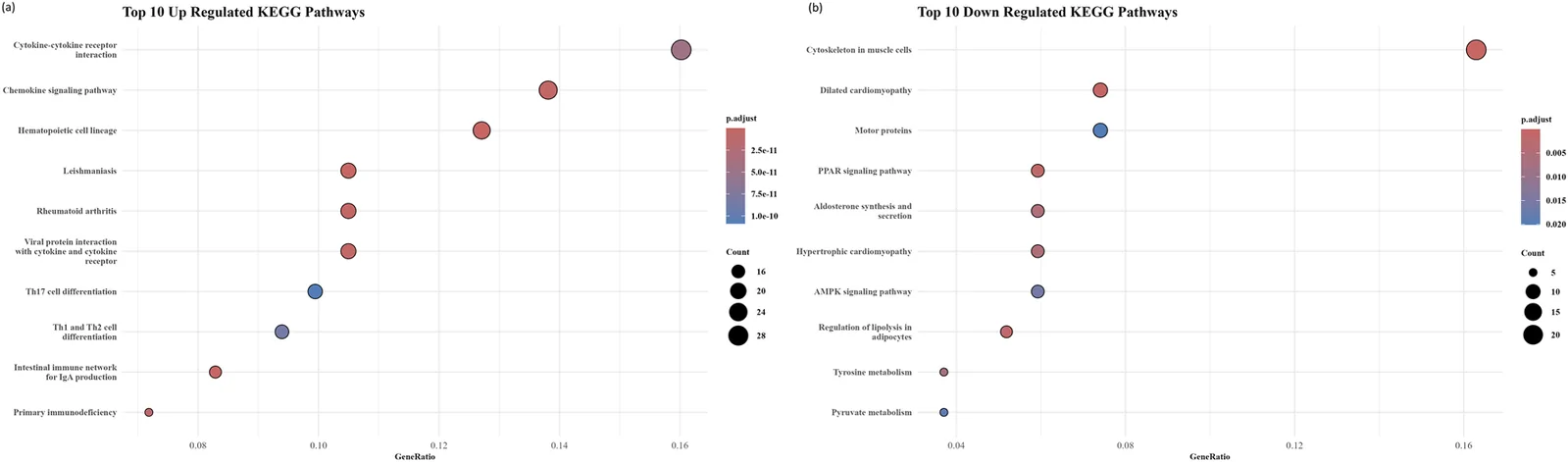
Enriched pathways of DEGs from Synovial tissues. **(a)** Upregulated pathways **(b)** Downregulated pathways.

**FIGURE 5:**
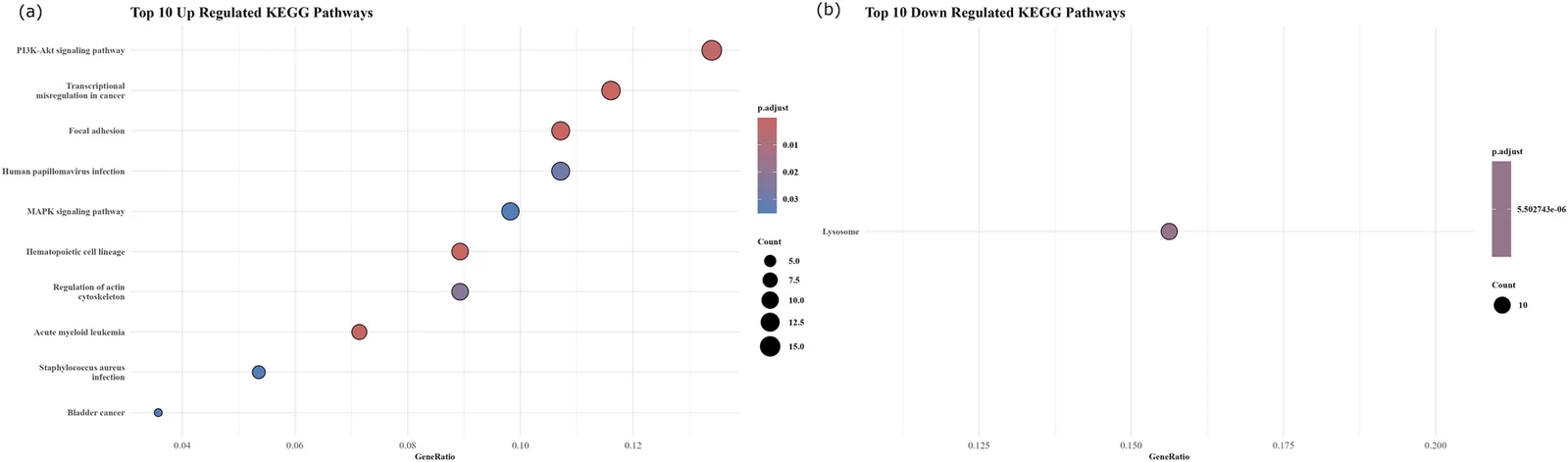
Enriched pathways of DEG from Synovial macrophages. **(a)** Upregulated pathways **(b)** Downregulated pathways.

### 3.4 Weighted gene co-expression network analysis of batch-corrected synovial tissue expression data

Batch correction was carried out before WGCNA analysis. The PCA revealed the presence of batch effects in the integrated dataset before the correction (Supplementary Figure S1A; Supplementary Figure S1B). The samples clustered predominantly based on the dataset origin rather than biological condition indicating technical variation is the major contributor for the overall expression differences. Plotting the samples based on the biological condition revealed substantial overlap, demonstrating that underlying biological variations were masked by inter-study heterogeneity. Following batch correction, the samples were observed to be mixed indicating successful decrease of technical variation. The biological condition plot also indicated improved arrangement of the samples within the principal component space, pointing towards improved detection of disease-related transcriptomics pattern after batch correction.

The PCA findings were further evaluated using PVCA (Supplementary Figure S1C and Supplementary Figure S1D). Before batch correction the batch effects contributed around 91.9% of the total variance, while batch-condition interaction accounted for 0.66%, biological condition contributed 0.04% and residual variation accounted for 7.39%. These results indicated that before correction technical differences dominated the integrated dataset. After the batch correction the variance contributed by the batch effects reduced to 82.3%. The contribution of biological condition increased to 4.0%, while the batch-condition interaction raised to 6.1%. The residual variance contributed about 8.0% of the overall variability.

Although ComBat correction reduced the batch-associated variance from 91.9% to 82.3%, PVCA indicated that batch effects remained the dominant source of variation in the integrated synovial tissue dataset. Therefore, the batch correction should be interpreted as partial attenuation rather than complete removal of technical heterogeneity. The continued contribution of batch effects may reflect differences in study design, microarray platforms, sample processing protocols, cohort composition, and other unmeasured technical factors across the public datasets. Consequently, downstream analyses based on the integrated synovial tissue dataset, including DEG detection, WGCNA module identification, and pathway enrichment, should be interpreted cautiously. We therefore consider these analyses exploratory and hypothesis-generating, rather than definitive evidence of disease-specific transcriptional regulation. Hence, validation in larger, platform-matched and independently generated synovial tissue cohorts is emphasized.

Despite correction, batch effects continued to drive most of the observed variation. ComBat is designed to reduce systematic technical bias while preserving biological variation and generally does not eliminate all inter-study differences. Complete removal of batch-associated variance may also risk overcorrection and inadvertent loss of biologically relevant information. Although residual batch effects persisted, both PCA and PVCA (Supplementary Figure S2) demonstrated a clear shift toward increased biological signal following correction. This increased condition-specific variance and reduced batch-driven segregation supported the use of the batch-corrected expression matrix for exploratory downstream analyses, while recognizing that residual batch effects could still influence part of the observed signal.

WGCNA constructs gene networks based on co-expression patterns to identify functional gene modules involved in disease pathogenesis. In the current study, WGCNA was performed using the batch-corrected integrated synovial tissue expression matrix generated from five datasets (Supplementary Material 2). Among these turquoise and blue modules exhibited the strongest correlations with disease traits, at 0.42 and 0.35, respectively. Further, to explore the turquoise module, we evaluated genes based on GS and MM. GS quantifies the relationship between individual genes and clinical traits, whereas MM represents the correlation between a gene and its corresponding module eigengene (module’s principal component). Elevated GS indicates disease relevance and high MM reflects a strong association with the module itself (Figure 6). Interestingly, the turquoise module demonstrated an atypical inverse correlation between these two metrics: genes with high GS scores generally corresponded with low MM scores, and *vice versa*. High-GS Genes (Disease-Relevant): Genes with high GS scores include *Glutathione Peroxidase 3* (*GPX3), KH RNA Binding Domain Containing, Signal Transduction Associated 3 (KHDRBS3), Transforming Growth Factor Beta Receptor 3 (TGFBR3), A-Kinase Anchoring Protein (AKAP1), SAM and SH3 Domain Containing 1(SASH1), Sorbin and SH3 Domain Containing 2 (SORBS2), Gram Domain Containing 1B (GRAMD1B), Nuclear Factor I C (NFIC), GULP PTB Domain Adapting Protein 1 (GULP1)*, and *NOVA Alternative Splicing Regulator 1* (*NOVA1)*. Among these, *GPX3* showed the highest GS score of 0.39. *GPX3*, part of the glutathione peroxidase family, plays a role in reducing extracellular Reactive Oxygen Species (ROS) levels, which protects host cells from oxidative damage, while oxidative stress is a known contributor to joint damage in RA. GPX3 has been previously linked to ROS regulation in neutrophils in autoimmune diseases like RA, systemic lupus erythematosus, and adult-onset still’s disease (Chen et al., 2023). *TGFBR3* (GS = 0.37), another high-GS gene, is a co-receptor for the *TGF-β* family and influences inflammatory cells within the synovium, which is a key site of inflammation in RA (Gonzalo-Gil and Galindo-Izquierdo, 2014).

**FIGURE 6:**
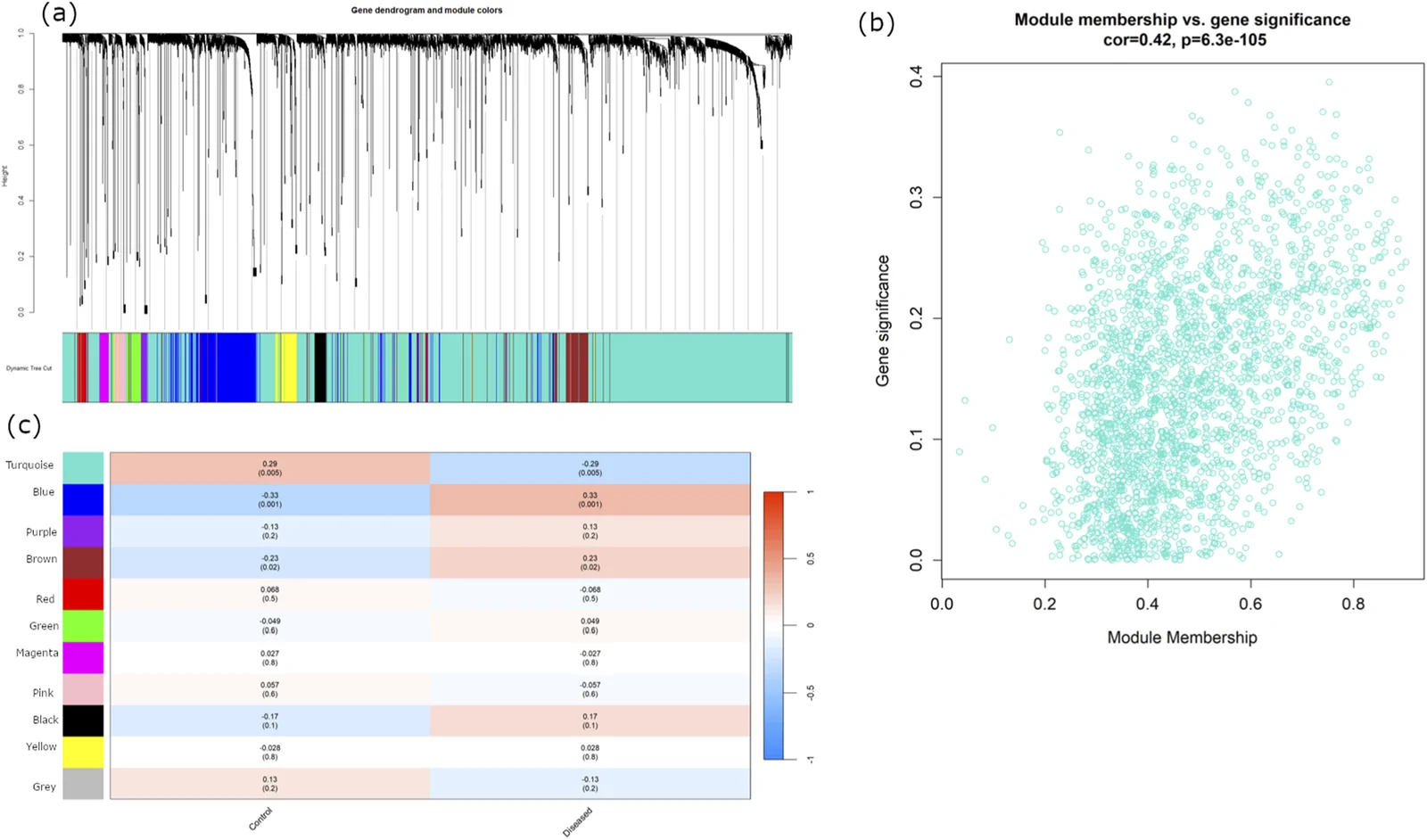
WGCNA and identification of hub candidates from batch-corrected synovial tissue expression data. **(a)** Gene clustering dendrogram representing color-coded modules In the diagram, each colour corresponds to a distinct co-expression module, while each branch represents an individual gene. **(b)** A scatter plot depicts the relationship between gene relevance and its inclusion within the turquoise gene module. **(c)** A heatmap visualizes the relationships between modules and traits, highlighting a strong correlation between the turquoise module and RA.

High-MM Genes (Module-Central): Genes with high MM, indicating strong connectivity within the turquoise module, include *SEL1L Family Member 3* (*SEL1L3), C-X-C Motif Chemokine Ligand 13 (CXCL13), T Cell Receptor Beta Constant 1 (TRBC1), C-C Motif Chemokine Receptor 5 (CCR5), CD2, CD3 Delta Subunit* (of the T cell Receptor Complex) *(CD3D), ADAM-Like Decoy Peptidase 1 (ADAMDEC1), CD27,* WGR Domain containing 1 *Vesicular Overexpression- Presumptive Protein 1 (VOPP1)*, and *Coronin 1A* (*CORO1A)*. SEL1L3 had the highest MM score of 0.9 which is a component of the endoplasmic reticulum-associated degradation pathway. The pathway helps to degrade the misfolded or abnormal proteins. RA is characterized by cellular stress and chronic inflammation, where SEL1L3 can aid in the mitigation of ER stress, leading to reduction of pro-inflammatory signals (Rahmati et al., 2018; Sun et al., 2014). CXCL13 has an MM score of 0.89. The chemokine CXCL13 is a marker of RA severity and plays a role in immune cell organization within the synovium (Achudhan et al., 2024; Bechman et al., 2020). The presence of high GS and MM genes highlights two distinct, interconnected features of RA pathogenesis. High-GS genes might be more useful as direct therapeutic targets, high-MM genes could help to stabilize cellular environments and reduce chronic stress responses, thus mitigating long-term RA progression.

### 3.5 Weighted gene co-expression network analysis–synovium macrophages

Prior to batch correction, PCA showed clear separation of the samples based on dataset along the PC1, which attributes to the 62.2% of total variance. The strong separation indicates dataset-specific technical variation (Supplementary Figure S3). When the samples were plotted based on biological conditions, there was also a clear segregation. However, the substantial influence of batch effects suggested that technical variation could potentially confound the interpretation of disease-associated expression patterns. Following the batch correction, the proportion of variance attributed by PC1 was reduced to 20.8% while PC2 increased to 13.3%, indicating a redistribution of variance following removal of systematic batch-associated effects. Samples were no longer separated predominantly by dataset origin, suggesting successful attenuation of batch-related variability. At the same time, separation between RA and normal samples remained evident, demonstrating that ComBat preserved biologically relevant transcriptional differences while reducing technical bias. PVCA showed that batch effects were the primary source of variation before correction, accounting for approximately 79% of the total variance, compared with 12% for biological condition and 7% for the batch-condition interaction (Supplementary Figure S4). After ComBat correction, batch-associated variance was effectively eliminated (∼0%), while the contributions of biological condition (∼40%) and batch-condition interaction (∼16%) increased, indicating successful removal of technical effects and improved representation of biological variation.

The batch-corrected merged synovial macrophage dataset was analysed using WGCNA. The analysis identified 26 modules (Figure 7), with the red module showing the strongest correlation with the disease trait (correlation = 0.95) (Supplementary Material 3). The red module showed genes with high GS (GS ≥ 0.5, n = 1,313) and MM (MM ≥ 0.7, n = 692) indicating that these genes not only show strong association with the disease but also show high connectivity within the module. To identify most crucial genes, we identified genes with both GS and MM > 0.9, yielding a list of 88 key genes.

**FIGURE 7:**
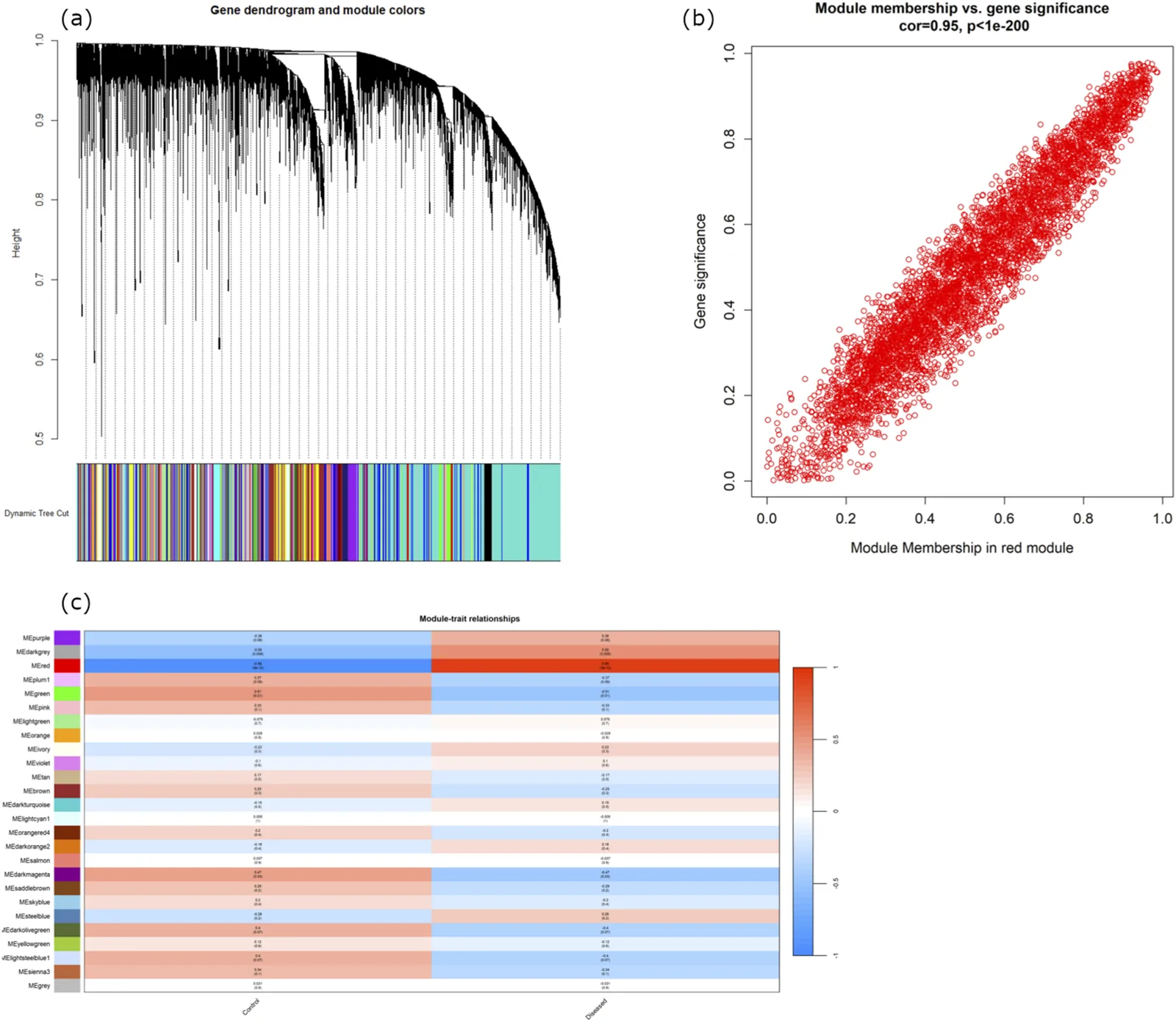
Analysis of WGCNA and identification of hub candidates for RA from synovial macrophages. **(a)** Cluster dendrogram showing co-expressed modules. **(b)** A scatter plot showing gene relevance in red module. **(c)** Heatmap showing relationships between modules and RA.

*S100 Calcium Binding Protein A10 (S100A10)* is the top-ranking gene with a GS of 0.97 and MM of 0.96. S100A10 is involved in the plasminogen activation system, which is crucial for macrophage migration in inflammatory response. *S100A10* gene protein allows assembly of plasminogen and its activators on the cell surface, thereby leading to macrophage migration (O’Connell et al., 2010). We also observed presence of other members of *S100* family genes, which includes *S100 Calcium Binding Protein A8 (S100A8)* and *S100 Calcium Binding Protein A12 (S100A12)*, with high GS and MM, indicating their importance in RA pathogenesis. S100A12 is a myeloid protein initially expressed in neutrophils and mononuclear macrophages, involved in inflammatory responses (Wu et al., 2022). Annexin A1 (ANXA1) is a calcium dependent phospholipid binding protein also showed high GS and MM values. *ANXA1* gene plays a important role in regulating innate immune responses, along with modulation of neutrophil adhesion and migration. Hence, it controls the spread of inflammation within the synovium (Han et al., 2020; Kelly et al., 2022).

We also observed three Fc Gamma Receptors (FCGR) namely, FCGR1BP, FCGR1A, and FCGR1CP, which are associated with bone erosion in inflammatory arthritis (Zuo and Deng, 2021). Additionally, we also identified other gene families, namely, *Heat Shock Proteins* (HSP) (*HSPA6, HSPA7*) and *Kruppel-like factors* (KLF) (*KLF4, KLF6,* and *KLF10*) that promote inflammatory arthritis by altering cytokine expression (Fouani et al., 2022) and apoptosis pathways respectively (Choi et al., 2018; Wang et al., 2022). *Cyclin Dependent Kinase Inhibitor 1A* (*CDKN1A)* and *KLF4* genes showed high MM and GS scores, indicating their role in driving synovial fibroblast proliferation and survival. Ras- Related C3 Botulinum Toxin Substrate 2 *(RAC2)*, a GTPase that promotes cell movement and Reactive Oxygen Species (ROS) production, which can draw more immune cells into joints and aggravates inflammation. Furthermore, higher expression of *SERPINA1* gene, which is a protease inhibitor, confirms its role in protecting the tissues from proteolytic enzyme-mediated damage in RA. High GS and MM values of genes of red module show a complex interplay between immune activation, cellular stress responses, and pro-inflammatory signalling in RA. RA is a multifactorial disease, driven by immune dysregulation, chronic inflammation, and oxidative stress mediated by the DEG of red modules emphasizing that these genes can be potential candidate biomarkers for further investigation. To assess their therapeutic relevance further functional validation is required.

### 3.6 Protein-protein interaction network of turquoise and red modules

The PPI networks of the turquoise and red modules were constructed and visualized (Figure 8). Hub genes were identified using the cytohubba plugin based on various topological metrics such as stress, radiality, Edge Percolated Component (EPC), Maximum Neighbourhood Component (MNC), closeness, betweenness, and degree. We selected list of hub genes based on MM and GS values and subsequently evaluated the roles of the most prominent genes in RA (Tables 2 and 3). The most prominent genes include CD markers like *CD86* (MM = 0.77), *CD8A* (MM = 0.71), *CD4* (MM = 0.70), *CD45* (*PTPRC*) (MM = 0.78), and *CD247* (MM = 0.80). These genes are implicated as potential contributors for synovial inflammation in RA patients. *CD86* was identified as top hub gene, critical costimulatory molecule found on Antigen-Presenting Cells (APCs). By binding to the CD28 receptor, CD86 drives the essential activation and survival of T cells. This interaction plays role in amplifying the immune response, contributing to the chronic inflammation in RA. In consistent with prior research, the pivotal role of CD86 in T-cell activation underscores its value as a target for autoimmune disease treatments (Thomas and Quinn, 1996). CD8^+^ T cells exert cytotoxic effects that indirectly regulate inflammatory processes by secreting cytokines or interactions with CD4^+^ T cells (Xiong et al., 2022). The inflammatory subsets of CD4^+^ T-cells, Th1, and Th17, leads to the release of pro-inflammatory cytokines like IFN-γ and IL-17, which exacerbate inflammation in RA. These results confirm the role of CD4^+^ T cells in autoimmune disorders, leading to the disruption of the inflammatory cascade. *CD45* (*PTPRC*) was also identified as a hub gene. The gene regulates neutrophils level in arthritic joints, indicating neutrophil-driven inflammation in RA patients (Kwok et al., 2012). *CD247* (CD3ζ) gene is involved in T-cell activation and signalling. Any change in *CD247* gene expression will cause T-cell dysfunction which may lead to autoimmune diseases. Downregulation of *CD247* gene was observed in most of the RA patients indicating deficiencies in T-cell responses, which may be responsible for disease progression and symptom severity (Mohammed et al., 2019). These findings help us to use CD247 can be a candidate biomarker which requires further investigation. However, its therapeutic relevance in RA cannot be inferred from transcriptomic analysis alone. The hub genes identified from the turquoise module suggest the DEGs involved in various inflammatory processes, which plays a significant role in driving disease pathogenesis.

**FIGURE 8:**
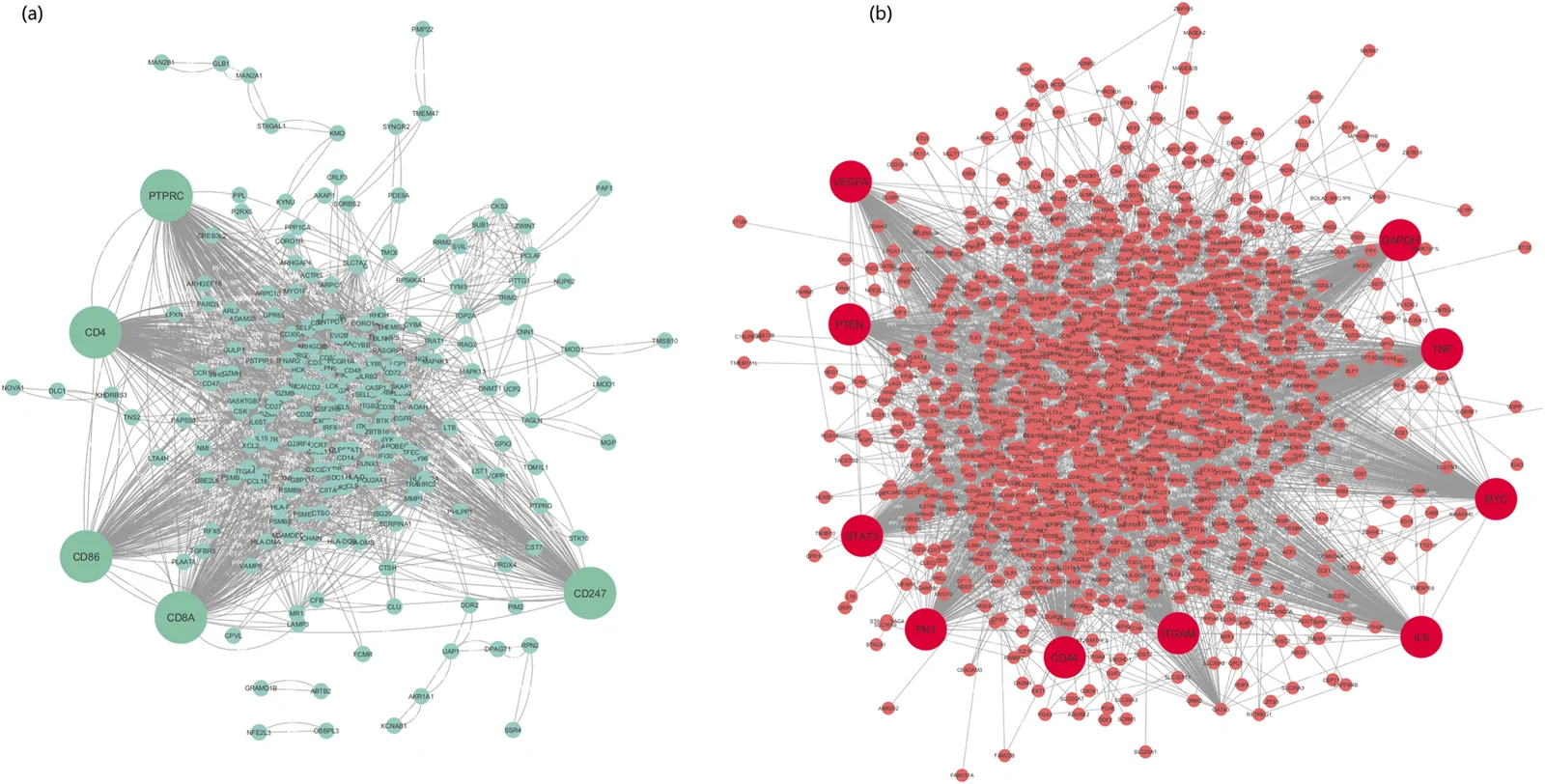
Protein-Protein interaction network **(a)** Turquoise module **(b)** Red module.

**TABLE 2:** Ranked genes by topological metrics of turquoise module.

Stress	Radiality	EPC	MNC	Closeness	Betweenness	Degree
CD86	CD86	CD86	IRF8	CD86	RAC2	IRF8
GLB1	CD8A	CD8A	CD86	BTK	CD86	CD86
ST6GAL1	ITGB2	ITGB2	CD8A	CD8A	CD8A	CD8A
MAN2A1	LCK	LCK	IL10RA	ITGB2	ENTPD1	IL10RA
DDR2	CD4	CD4	ITGB2	LCK	DDR2	ITGB2
DPAGT1	EGFR	CD2	CD4	CD4	KYNU	CD4
KYNU	VAV1	PTPRC	CD247	SYK	CD4	CD247
KMO	SYK	IL2RG	CCR5	CD247	EGFR	CCR5
EGFR	CD247	SELL	PTPRC	PTPRC	PTPRC	PTPRC
UAP1	PTPRC	GZMB	SELL	SELL	UAP1	SELL

**TABLE 3:** Ranked genes by topological metrics of red module.

Stress	Radiality	EPC	MNC	Closeness	Betweenness	Degree
CDC42	MYC	CD44	CD44	MYC	CDC42	CD44
MYC	PTEN	MYC	MYC	PTEN	MYC	MYC
PTEN	RHOA	ITGAM	PTEN	RHOA	PTEN	PTEN
RHOA	CCND1	FCGR2C	ITGAM	CCND1	RHOA	ITGAM
IL6	IL6	IFNG	IL6	IL6	APP	IL6
STAT3	STAT3	IL6	STAT3	STAT3	IL6	STAT3
FN1	FN1	STAT3	FN1	FN1	FN1	FN1
UBC	VEGFA	VEGFA	VEGFA	VEGFA	UBC	VEGFA
TNF	TNF	TNF	TNF	TNF	TNF	TNF
GAPDH	GAPDH	GAPDH	GAPDH	GAPDH	GAPDH	GAPDH

Topological metrics analysis of the red module showed *MYC Proto-Oncogene* (*MYC)*, *Interleukin-6* (*IL-6)*, *Tumor Necrosis Factor* (*TNF)*, *Glyceraldehyde-3-Phosphate Dehydrogenase* (*GAPDH)*, *Phosphatase and Tensin Homolog* (*PTEN)*, *Signal Transducer and Activator of Transcription 3* (*STAT3)*, *Fibronectin 1* (*FN1)*, *Vascular Endothelial Growth Factor A* (*VEGFA)*, *CD44*, and *Integrin Subunit Alpha M* (*ITGAM)* genes as top players of the module. All these hub genes of the red module have important role in controlling inflammatory cytokines, immune cell signalling, and tissue remodelling. Cytokines IL6 and TNF were involved in activation of inflammatory pathways that may lead to immune cell infiltration and joint damage in RA patients (Jang et al., 2021; Jarlborg and Gabay, 2022). Similarly, by activating STAT3*,* cytokines like IL-6 and TNF-α promote inflammatory signalling that may help in synovial cell survival within the inflamed synovium, thereby aggravating disease progression (Isomäki et al., 2015; Murakami and Hirano, 2012; Oike et al., 2017). Proteins such as FN1, GAPDH, and CD44 are involved in maintaining cellular structure, metabolism and tissue remodelling, the dysregulation of these genes reveals the importance of structural and metabolic modification within the RA synovium. FN1 and CD44 proteins enable inflammatory cells to persist in the synovium by enhancing cell adhesion and tissue invasion properties, these changes contribute to chronic inflammation. Recent studies also revealed that elevated level of CD44 Variant 9 (CD44v9) in synovial macrophages of RA patients, underscores the involvement of CD44 in the development and progression of the disease (Naor and Nedvetzki, 2003; Qadri, 2023). Elevated *GAPDH* gene expression signifies a metabolic shift within synovial cells, aiding the high-energy requirements of proliferative and inflammatory processes (Montero-Melendez and Perretti, 2014).

Furthermore, *MYC* and *PTEN* genes emphasize connectivity between immune modulation, cell proliferation, and apoptosis. In RA, MYC’s role in cell proliferation and PTEN-mediated regulation, jointly influence synovial cell hyperplasia and aberrant immune cell activity. The downregulation of PTEN in synovial fibroblasts, predominantly in the areas of invasive growth and tissue destruction, highlights a pivotal pathogenic between fibroblasts and macrophages in RA. In the RA synovium, macrophages mediated inflammatory cytokines, particularly TNF and IL-6, which not only drive inflammation but also trigger the downregulation of PTEN in adjacent fibroblasts. Consequently, the altered fibroblasts create a microenvironment for macrophage retention and activation, that leads to inflammation and tissue destruction. Furthermore, PTEN downregulation triggers synovial fibroblasts to release additional cytokines and chemokines, which attract more macrophages to the joint and increases local joint (Pap et al., 1999; 2000). Similarly, upregulation of VEGFA promotes angiogenesis in RA. This neovascularization within the synovium not only sustains oxygen supply and nutrients to inflamed tissues but also facilitates immune cell infiltration, thereby maintaining the inflammatory state in RA joints (Taylor, 2002). Overall, the key hub genes identified act as critical regulatory nodes, controlling inflammation, cell survival, and synovial tissue remodelling. These insights will clarify potential treatment strategies, such as targeting cytokine pathways (e.g., IL-6, TNF) or adjusting metabolic and angiogenic processes (e.g., GAPDH, VEGFA). Understanding these molecular mechanisms in RA could facilitate the development of various targeted therapies to reduce inflammation and to prevent joint damage in diseased patients.

Biomarkers from the red module, specifically identified from synovial macrophages, can be ideal than the broader synovium (turquoise module) because they more precisely mediate of RA. Synovial macrophages serve as key drivers of RA pathology by secreting pro-inflammatory cytokines like IL-6 and TNF which directly contribute to joint destruction. Synovial macrophage biomarkers offer precise indicators of inflammation, as they correlate well with disease severity, and align closely with therapeutic targets, as many RA treatments focus on reducing macrophage activity. Additionally, the red module genes exhibit high gene significance and module membership values above 0.9, indicating a strong association with disease traits, which enhances their reliability for monitoring RA activity and treatment response.

### 3.7 Biomarker selection and validation

The red module genes, previously identified as relevant to RA pathogenesis were selected as features, and the datasets were filtered to include only these genes. SVM and RF models were trained and tuned to optimize hyperparameters, and important genes were extracted using Recursive Feature Elimination (RFE) for SVM and feature importance scores for RF. A predefined feature-importance cutoff was not applied during the Random Forest (RF) analysis. Instead, all 88 candidate genes identified from the WGCNA red module were ranked according to their RF importance scores (Mean Decrease Gini), The final candidate biomarkers were identified by integrating the results of RF and SVM-RFE, with consensus genes defined as those top six genes consistently selected by both feature-selection methods (Table 4). The identified six genes are computationally prioritised candidate biomarkers and not completely experimentally validated. Six consensus biomarkers, namely, Interferon Gamma (*IFNG),* Phosphatase and Tensin Homolog *(PTEN),* Interleukin 2 Receptor Subunit Gamma *(IL2RG),* Cyclin Dependent kinase inhibitor 2D *(CDKN2D),* Vascular Endothelial Growth Factor *(VEGFA),* and Cyclin Dependent Kinase Inhibitor 1A (*CDKN1A*) were identified as genes consistently ranked as predictive by both models. The identified consensus genes are critical to RA pathogenesis, encompassing inflammation (*IFNG*, a pro-inflammatory cytokine involved in T-cell responses; *IL2RG*, essential for cytokine signaling), cell cycle regulation (*CDKN2D* and *CDKN1A*), which mediate cell cycle arrest affecting immune cell proliferation and tissue remodelling), and vascular remodelling (*VEGFA*, driving angiogenesis and synovial inflammation). *PTEN*, a tumour suppressor, influences RA by regulating inflammatory control and synovial fibroblast activity.

**TABLE 4:** Final six biomarkers identified by SVM-RFE and RF analyses.

Gene	SVM-RFE score	RF importance score
IFNG	0.170889	0.189818
PTEN	0.165556	0.182909
IL2RG	0.153778	0.181455
CDKN2D	0.183333	0.180364
VEGFA	0.162222	0.163818
CDKN1A	0.186000	0.163546

Validation of SVM and RF models was done using three test sets: macrophage expression (Test Sets 1 and 2) and combined synovial tissue expression (Test Set 3). To provide a more comprehensive assessment of predictive performance, models were evaluated using accuracy, sensitivity, specificity, precision, F1-score, AUC, and 95% confidence intervals, rather than accuracy alone. The sample size and class distribution of each training and testing dataset are reported in Supplementary Table S1 and the complete classification performance metrics are summarized in Table 5. Using the six-gene signature, both SVM and Random Forest (RF) achieved perfect performance on Test Set 1, with 100% accuracy, sensitivity, specificity, F1-score, and AUC. However, performance varied considerably across the other validation datasets. However, because GSE10500 contributed to biomarker discovery, this result should not be interpreted as independent validation and likely represents an optimistic estimate of classifier performance. Therefore, the perfect performance in this dataset is reported only as a non-independent internal evaluation of model fit. On Test Set 2, SVM outperformed RF, achieving 75.0% accuracy and 100% sensitivity, whereas RF achieved only 25.0% accuracy and failed to identify any RA cases. Similarly, on the larger Test Set 3, SVM demonstrated moderate predictive performance with 68.0% accuracy, 79.5% sensitivity, 21.1% specificity, an F1-score of 0.80, and an AUC of 0.494, while RF showed poor classification performance with 19.6% accuracy, 0% sensitivity, and 100% specificity despite a higher AUC of 0.678. Therefore, the present results do not support the conclusion that SVM is a superior or more suitable diagnostic classifier for the six-gene signature. Instead, SVM showed greater RA-case sensitivity, while its AUC values in the independent external datasets indicate inadequate overall discriminatory ability. Thus, the machine-learning findings should be interpreted cautiously as exploratory biomarker-prioritization results rather than evidence of a validated diagnostic classifier (Figure 9). Despite classification performance, including perfect accuracy in some test sets, was observed, these results should be interpreted with caution because the same limited synovial macrophage datasets contributed to both feature selection and classifier construction. Therefore, the classification metrics may represent optimistic estimates and may not fully reflect performance in independent patient cohorts.

**TABLE 5:** Performance metrics of SVM and RF classifiers.

Model	Dataset	Accuracy	Sensitivity	Specificity	Precision	F1-score	AUC	AUC (Lower 95CI)	AUC (Upper 95CI)
SVM	Test set 1	1	1	1	1	1	1	1	1
	Test set 2	0.75	1	0	0.75	0.857	0.5	0.5	0.5
	Test set 3	0.68	0.795	0.211	0.805	0.8	0.494	0.359	0.628
RF	Test set 1	1	1	1	1	1	1	1	1
	Test set 2	0.25	0	1	NA	NA	0.5	0.5	0.5
	Test set 3	0.196	0	1	NA	NA	0.678	0.537	0.819F

NA, not available.

**FIGURE 9:**
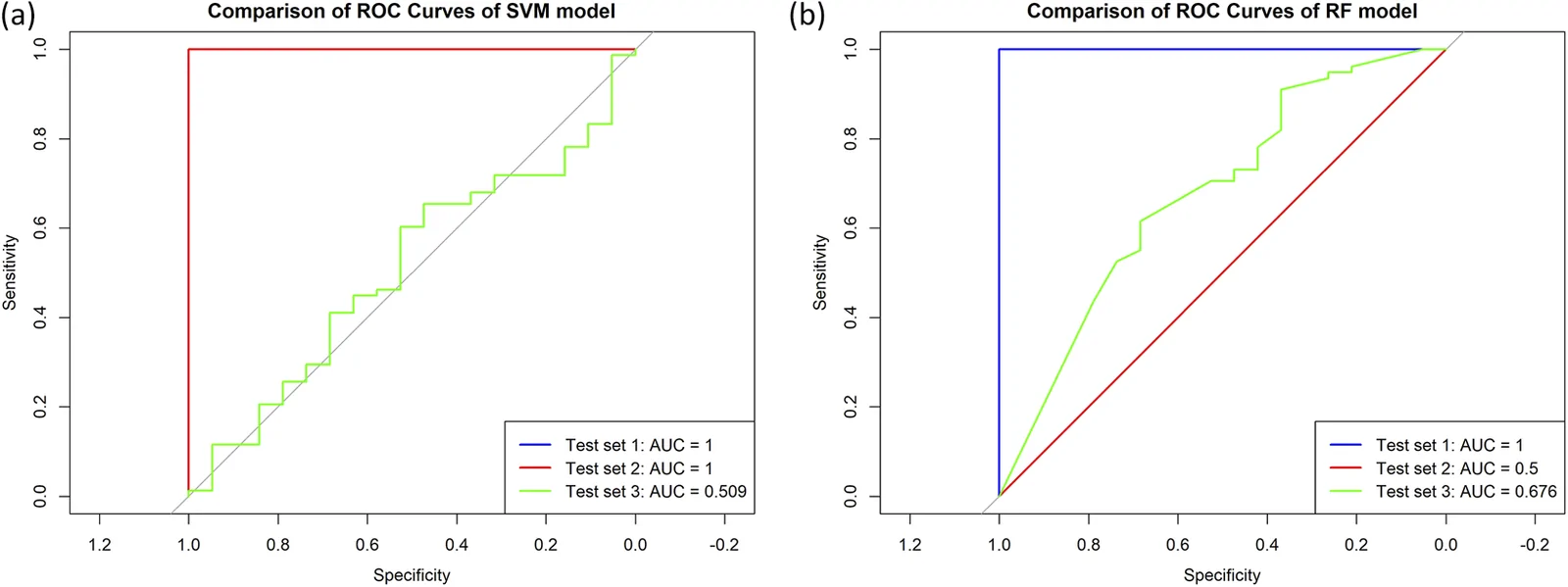
ROC analysis of six hub genes of three test sets. **(a)** SVM models and **(b)** RF models.

Our analysis prioritized consensus genes such as *IFNG* and *PTEN* analysis play critical roles in immune regulation and inflammatory responses, making high priority candidates for further investigation. These findings support the utility of SVM and RF for biomarker identification in RA and demonstrates the need for integrating data from diverse sources to achieve a comprehensive understanding of disease mechanisms. Due to the limited availability of synovial macrophage datasets, partially overlapping datasets were used during feature selection and classifier development. This may introduce information leakage and overfitting, causing the model to learn dataset specific patterns rather than generalizable RA associated signals. The perfect performance observed in the non-independent evaluation dataset is therefore likely to represent an inflated estimate of model performance. Moreover, the poor AUC values of SVM in the external test datasets indicate that the classifier did not reliably discriminate RA from control samples despite showing high sensitivity. Thus, the machine learning results should be considered exploratory and hypothesis generating rather than confirmatory. Future studies should apply nested cross-validation, perform feature selection strictly within each training fold, and validate the final biomarker panel in larger independent synovial macrophage cohorts. Functional studies, including gene knockdown, overexpression, and cytokine assays, are also required to confirm the mechanistic relevance of the prioritized genes in RA pathogenesis.

### 3.8 Experimental validation for biomarkers in serum samples

Computational analysis identified six RA-associated biomarkers (*IFNG*, *PTEN*, *IL2RG*, *CDKN2D*, *VEGFA*, *CDKN1A*). Among these, *IL2RG* and *IFNG* were selected for wet lab validation due to high GS and MM metrics (*IFNG* with GS-0.65 and MM-0.619 and *IL2RG* with GS-0.906 and MM-0.963) in the WGCNA red module, marking them as key hub nodes. Unlike intracellular cell-cycle inhibitors or tumor suppressors, IL2RG encodes the common gamma chain shared across multiple interleukin receptors (IL-2, IL-4, IL-7, IL-9, IL-15, and IL-21), while IFNG is a central pro-inflammatory cytokine actively driving T-cell and macrophage cascades. Given their direct roles in extracellular signaling, they represent ideal, biologically active targets for minimally invasive protein-level detection. Although, we focused our primary transcriptomic analysis on synovial tissue and macrophages due to their key roles in RA pathology, cross-dataset comparisons revealed systemic dysregulation of IL2RG and IFNG within peripheral blood and circulating immune cells. Continuous cellular trafficking and cytokine release from the synovium into the vascular compartment drive this effect, establishing a clear functional bridge between local joint transcription and the systemic circulation. Thus, quantifying serum IL2RG and IFNG level via ELISA directly validated our computational findings in a physiologically relevant context.

Serum concentrations of IL2RG and IFNG were measured via ELISA to compare RA patients against the control group. IL2RG expression levels were significantly elevated in RA patients (1.34 ± 0.11 ng/mL) compared to healthy controls (0.83 ± 0.07 ng/mL) (unadjusted *p* = 0.0003; Bonferroni-adjusted 0.0006) (Figure 10a). These values confirm a significant increase of IL2RG in the RA patients. The elevated expression of IL2RG signals underlying immune cell activation and dysregulation, which leads to chronic synovial inflammation. In addition, statistical analysis also indicated that IFNG expression levels were marginally elevated in RA patients (1.91 ± 0.06 pg/mL) compared to healthy controls (1.69 ± 0.06 pg/mL); unadjusted *p* = 0.0126, Bonferroni-adjusted *p* = 0.0252 (Figure 10b). Although these preliminary experiments show elevated serum levels of IL2RG and IFNG in RA patients compared to healthy controls, validating only these two biomarkers does not confirm the entire machine learning classifier. Consequently, additional functional assays and independent cohort studies are necessary to establish their diagnostic and mechanistic relevance to RA.

**FIGURE 10:**
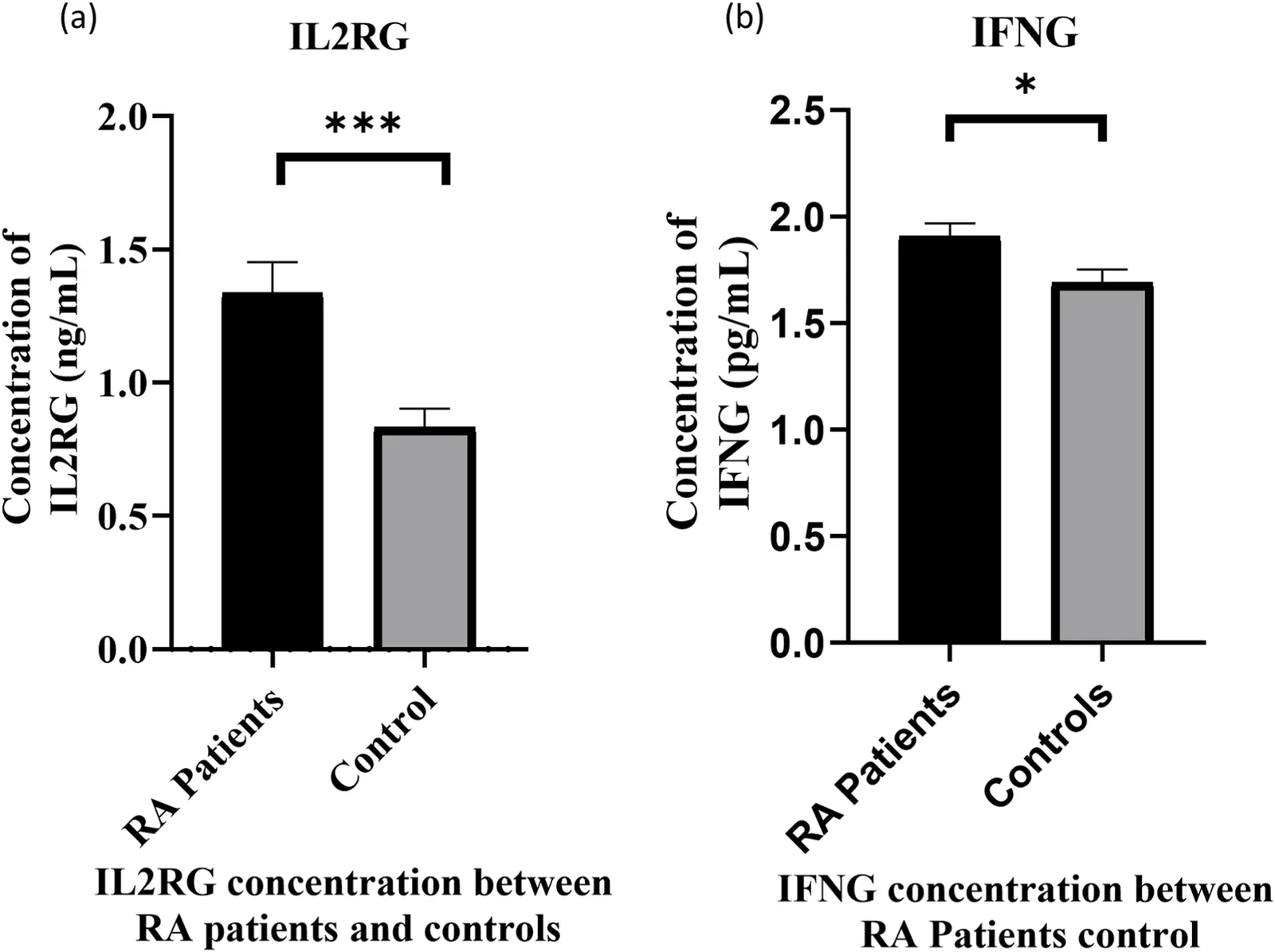
**(a)** IL2RG expression level comparison between RA patients and control serum samples; **(b)** IFNG expression level comparison between RA patients and control serum samples.

## 4 Conclusion

This study employed a combination of transcriptomic analysis and network approach to investigate the genetic drivers of RA. Analysis of DEGs across various cell types, synovial tissue and macrophages exhibited significant transcriptomic changes, emphasizing its importance for in-depth study. The functional enrichment, pathway analysis and WGCNA revealed key genes and pathways involved in RA pathogenesis. The machine learning analysis prioritized a six gene-candidate biomarker signatures (*IFNG, PTEN, IL2RG, CDKN2D, VEGFA,* and *CDKN1A*) specifically within synovial macrophages. Among these, IL2RG and IFNG were further supported by ELISA-based serum validation, showing significantly elevated levels in RA patients compared with healthy controls. However, the machine learning classifier results should be interpreted cautiously because partially overlapping datasets were used for feature selection and classifier development, and SVM showed AUC values close to 0.5 in the external evaluation datasets. Therefore, the six-gene panel should not be considered a validated diagnostic classifier at this stage. Instead, these genes represent computationally prioritized candidate biomarkers that require validation in larger independent cohorts and functional studies. Overall, the current study indicates that synovial macrophages are key mediators of RA pathogenesis and serve as a valuable source for identifying candidate biomarkers. However, further independent validation and functional characterization remain essential to determine whether these identified genes directly contribute to RA mechanisms or offer therapeutic relevance.

## Data Availability

The R scripts used for data processing, WGCNA analysis, for this work and/or its publication. machine learning feature selection, and classifier construction are available at: https://github.com/sudharsanabioinfo-afk/RA-biomarker-analysis.
