# Supplementary material for: Integrating transcriptomic analysis to explore biomarkers and therapeutic targets for rheumatoid arthritis: a case-control and systems biology approach
Source: Front Bioinform. 2026 Oct 5;6:1891543. doi: 10.3389/fbinf.2026.1891543 (PMC13677927; doi:10.3389/fbinf.2026.1891543)
Supplement: Supplementary file 1 [file DataSheet1.zip › Supplementary Table 2.docx]

**Supplementary Table 2: Number of differentially expressed genes across different datasets**

| **GEO Accession** | **Sample Type** | **Number of up-regulated genes** | **Number of down-regulated genes** |
| --- | --- | --- | --- |
| GSE10500 | Synovial macrophages | 465 | 476 |
| GSE97779 | Synovial macrophages | 1019 | 886 |
| GSE65010 | T Cells | No significant genes found | |
| GSE93776 | CD4+ T Cells | No significant genes found | |
| GSE56649 | CD4+ T Cells | 486 | 469 |
| GSE93776 | CD8+ T Cells | 0 | 2 |
| GSE93776 | NKT Cells | 0 | 1 |
| GSE93776 | NK Cells | 1 | 1 |
| GSE93776 | Neutrophil Cells | No significant genes found | |
| GSE93776 | Monocyte Cells | No significant genes found | |
| GSE38351 | Monocyte Cells | 5 | 6 |
| GSE71370 | CD14+ | 1 | 0 |
| GSE12021 | Synovial tissues | 191 | 194 |
| GSE1919 | Synovial tissues | 228 | 200 |
| GSE55235 | Synovial tissues | 472 | 447 |
| GSE55457 | Synovial tissues | 268 | 220 |
| GSE77298 | Synovial tissues | 170 | 117 |
| GSE15573 | Peripheral blood mononuclear cells | 15 | 1 |
| GSE17755 | Peripheral blood mononuclear cells | 19 | 5 |
| GSE68689 | Whole blood | No significant genes found | |
| GSE100191 | Peripheral blood mononuclear cells | No significant genes found | |
| GSE93272 | Whole blood | No significant genes found | |
| GSE110169 | Peripheral blood mononuclear cells | No significant genes found | |
