# Supplementary material for: Integrating transcriptomic analysis to explore biomarkers and therapeutic targets for rheumatoid arthritis: a case-control and systems biology approach
Source: Front Bioinform. 2026 Oct 5;6:1891543. doi: 10.3389/fbinf.2026.1891543 (PMC13677927; doi:10.3389/fbinf.2026.1891543)
Supplement: Supplementary file 1 [file DataSheet1.zip › Supplementary Table 1.docx]

**Supplementary Table 1: Gene expression datasets used in the current study**

| **GEO Accession** | **Sample Type** | **Array Type** |
| --- | --- | --- |
| GSE10500 | Synovial macrophages | Affymetrix HG_U95Av2 |
| GSE97779 | Synovial macrophages | Affymetrix [HG-U133_Plus_2] |
| GSE65010 | T cells | Affymetrix [HG-U133_Plus_2] |
| GSE93776 | CD4+ T cells | Affymetrix [HG-U133_Plus_2] |
| GSE56649 | CD4+ T cells | Affymetrix [HG-U133_Plus_2] |
| GSE93776 | CD8+ T cells | Affymetrix [HG-U133_Plus_2] |
| GSE93776 | NKT cells | Affymetrix [HG-U133_Plus_2] |
| GSE93776 | NK cells | Affymetrix [HG-U133_Plus_2] |
| GSE93776 | Neutrophil cells | Affymetrix [HG-U133_Plus_2] |
| GSE93776 | Monocyte cells | Affymetrix [HG-U133_Plus_2] |
| GSE38351 | Monocyte cells | Affymetrix HG-U133A |
| GSE71370 | CD14+ | Affymetrix [HG-U133_Plus_2] |
| GSE12021 | Synovial tissues | Affymetrix [HG-U133A] |
| GSE1919 | Synovial tissues | Affymetrix [HG_U95A] |
| GSE55235 | Synovial tissues | Affymetrix [HG-U133A] |
| GSE55457 | Synovial tissues | Affymetrix [HG-U133A] |
| GSE77298 | Synovial tissues | Affymetrix [HG-U133_Plus_2] |
| GSE15573 | Peripheral blood mononuclear cells | Illumina human-6 v2.0 |
| GSE17755 | Peripheral blood mononuclear cells | Hitachisoft AceGene Human Oligo Chip |
| GSE68689 | Whole blood | Affymetrix HuGene-1_0-st |
| GSE100191 | Peripheral blood mononuclear cells | Agilent-026652 |
| GSE93272 | Whole blood | Affymetrix HG-U133_Plus_2 |
| GSE110169 | Peripheral blood mononuclear cells | AffymetrixHG-U219 |
