# Supplementary material for: Integrating transcriptomic analysis to explore biomarkers and therapeutic targets for rheumatoid arthritis: a case-control and systems biology approach
Source: Front Bioinform. 2026 Oct 5;6:1891543. doi: 10.3389/fbinf.2026.1891543 (PMC13677927; doi:10.3389/fbinf.2026.1891543)
Supplement: Supplementary file 2 [file DataSheet2.zip › Supplementary figure Presentation/Supplementary Files captions.docx]

**Supplementary File 1** Differentially expressed genes (DEG) expressed in different cell types

**Supplementary File 2** Gene significance and Module membership of Turquoise module

**Supplementary File 3** Gene significance and Module membership of Red module
